# Computational study on new natural compound agonists of stimulator of interferon genes (STING)

**DOI:** 10.1371/journal.pone.0216678

**Published:** 2019-05-23

**Authors:** Sheng Zhong, Weihang Li, Yang Bai, Bo Wu, Xinhui Wang, Shanshan Jiang, Yingjing Zhao, Jiaxin Ren, Hui Li, Rihua Jin

**Affiliations:** 1 Department of Neurosurgery, the First Hospital of Jilin University, Changchun, China; 2 Department of Surgery, Brigham and Women’s Hospital, Harvard Medical School, Boston, the United States of America; 3 Clinical College, Jilin University, Changchun, China; 4 Department of Orthopedics, the First Hospital of Jilin University, Changchun, China; 5 Department of Oncology, the First Hospital of Jilin University, Changchun, China; 6 College of pharmacy, Jilin University, Changchun, China; Brooklyn College of the City University of New York, UNITED STATES

## Abstract

**Objective:**

This study aimed to screen lead compounds and medication candidates from drug library (ZINC database) which has potential agonist effect targeting STING protein.

**Methods and materials:**

A series of computer-aided virtual screening techniques were utilized to identify potential agonists of STING. Structure-based screening using Libdock was carried out followed by ADME (absorption, distribution, metabolism, excretion) and toxicity prediction. Molecular docking was performed to demonstrate the binding affinity and mechanism between ligands and STING dimers. Molecular dynamic simulation was utilized to evaluate the stability of ligand-receptor complex. Finally, animal experiment was conducted to validate the effectiveness of selected compounds.

**Results:**

Three novel natural compounds 1,2,3 (ZINC000015149223, ZINC000011616633 and ZINC000001577210, respectively) from the ZINC15 database were found binding to STING with more favorable interaction energy. Also, they were predicted with less ames mutagenicity, rodent carcinogenicity, non-developmental toxic potential and tolerant with cytochrome P450 2D6 (CYP2D6). The ligand chemical structure analysis showed the three compounds were inborn axisymmetric, such chemical structures account for combining and activating process of STING protein dimers. The dynamic simulation analysis demonstrated that ZINC000015149223-, ZINC000011616633- and ZINC000001577210-STING dimer complex had more favorable potential energy compared with amidobenzimidazole (ABZI) and they can exist in natural environments stably. Animal experiments also demonstrated that these three compounds could suppress tumor growth.

**Conclusion:**

This study demonstrates that ZINC000015149223, ZINC000011616633 and ZINC000001577210 are potential agonists targeting STING protein. These compounds are safe drug candidates and have a great significance in STING agonists development.

## Introduction

Stimulator of interferon genes (STING) is a receptor in the endoplasmic reticulum that propagates innate immune sensing of cytosolic pathogen derived- and self-DNA [1]. STING is a 378 amino acid protein, which mainly contains three structural domains: they are N-terminal transmembrane domain (aa 1–154), central globular domain (aa 155–341), and C-terminal tail (aa 342–379). Basically, STING can form symmetrical dimers combined with its ligands in V-shaped conformation and it doesn’t completely cover the bound ligands [2]. A natural STING agonist, cGAMP, can bound into pocket region of STING. The cytoplasmic facing C-terminal domain of STING is a homodimeric complex which interacts with cGAMP through a network of hydrogen bonds and water-mediated interactions within a large (1,400 nm^3^) binding pocket [3–5]. STING plays a crucial role in various diseases, inactivation of cGAS-cGAMP-STING function is reported to be associated with many severe diseases such as cancer, obesity, liver injury, sugar-lipid metabolism and virus infection and etc [6–8]. However, STING activation process is easily to be inhibited in some severe diseases conditions, such as cancer, viral infection [9]. It will finally results in the inactivation of STING pathway. Therefore, screening and designing potent STING agonists is of great importance for cancer immune therapy and other infectious diseases treatment.

Exogenous or autogenous DNA accumulation in the cytoplasm can lead to a strong immune response. Increasing evidence has suggested an important interaction between tumor DNA damage and immune system during oncogenesis [10]. Also, several publications suggest that cGAS-cGAMP-STING pathway play a significant role during cancer immune evasion and immune system stimulation process [11–13]. In this process, the cGAS-cGAMP-STING pathway is considered to play a significant role. Cytoplasmic free DNA, which is considered as a dangerous signal to body, is recognized by nucleotidyl transferase cGAS (DNA receptor ring GMP-AMP synthase) [14]. After cGAS is activated by double-stranded DNA (dsDNA), it will catalyze the synthesis of a noncanonical cyclic dinucleotide 2’5’-cGAMP from adenosine triphosphate (ATP) and guanosine triphosphate (GTP) [15]. Next, the downstream protein STING (stimulator of interferon genes), which acting as a hub mediating factor in cGAS-cGAMP-STING pathway, can be activated by either second messengers, such as cyclic adenosine phosphate (cAMP) and cyclic guanosine phosphate (cGMP), or cGAMP, which is produced by cGAS sensing cytoplasmic DNA. Tumor-derived DNA activates cGAS to produce cGAMP, the endogenous ligand of STING, resulting in downstream signaling cascade via recruitment of serine/threonine-protein kinase (TBK1), phosphorylation of the interferon regulatory transcription factor IRF3, and the production of type I interferon (IFN). Accumulate pro-inflammatory cytokines, type I interferon and other cytokines will finally lead to a correspondent immune response [16]. However, this immune signaling pathway is aberrantly suppressed in some specific cancer microenvironment, which finally lead to cancer immune evasion and oncogenesis [17]. On the other hand, in some specific exogenous bacterial or viral infection conditions, abnormal deposition of host DNA in cytosol can also activate the cGAS-cGAMP-STING signaling pathway cascade overwhelmingly, and it will result in uncontrolled over-inflammation, autoimmune diseases and immune cell draining [18], over-activation of STING contributes to even triggers the onset of autoimmune disorders such as systemic lupus erythematosus [19]. Therefore, there is an urgent need to develop a series of agonists and inhibitors targeting to STING.

Natural products and their derivatives possess unique chemical structures and have potential biological function, they have made a great contribution to medication design and refinement, they also represent the major part of current pharmaceutical market [20,21]. In recent years, there are several publications report that small molecule compounds have regulatory functions regarding to STING activity [22,23]. The purpose of this study is to determine lead compounds of STING agonist for drug development and compounds modification. This study employed a series of structural biological and chemistry method (including virtual screening, molecule docking and etc) to screen and identify the lead compounds which had potential regulatory functions to STING. At the same time, our study also predicted absorption, distribution, metabolism, excretion and toxicity of these compounds. This study provided a list of drug candidates and their pharmacological properties, which could provide a solid basis for STING agonists development research.

## Methods and materials

### Docking software and ligand library

Discovery Studio is a suite of software for simulating small molecule and macromolecule systems, which is developed aiming to screen, design and modify the potential drugs by structural chemical and structural biological computation, large amount of lead compounds and drug candidates were identified and refined through this method. Libdock and ADME (absorption, distribution, metabolism, excretion) modules of Discovery Studio 4.5 software (DS4.5, Accelrys, Inc) were employed for virtual screening. CDOCKER was used for docking study. The Natural Products (NP) database in the ZINC15 database was selected to screen STING agonists. ZINC15 database is a free database of commercially-available compounds provided by the Irwin and Shoichet Laboratories in the Department of Pharmaceutical Chemistry at the University of California, San Francisco (UCSF).

### Structure-based virtual screening using libdock

**Fig 1:**
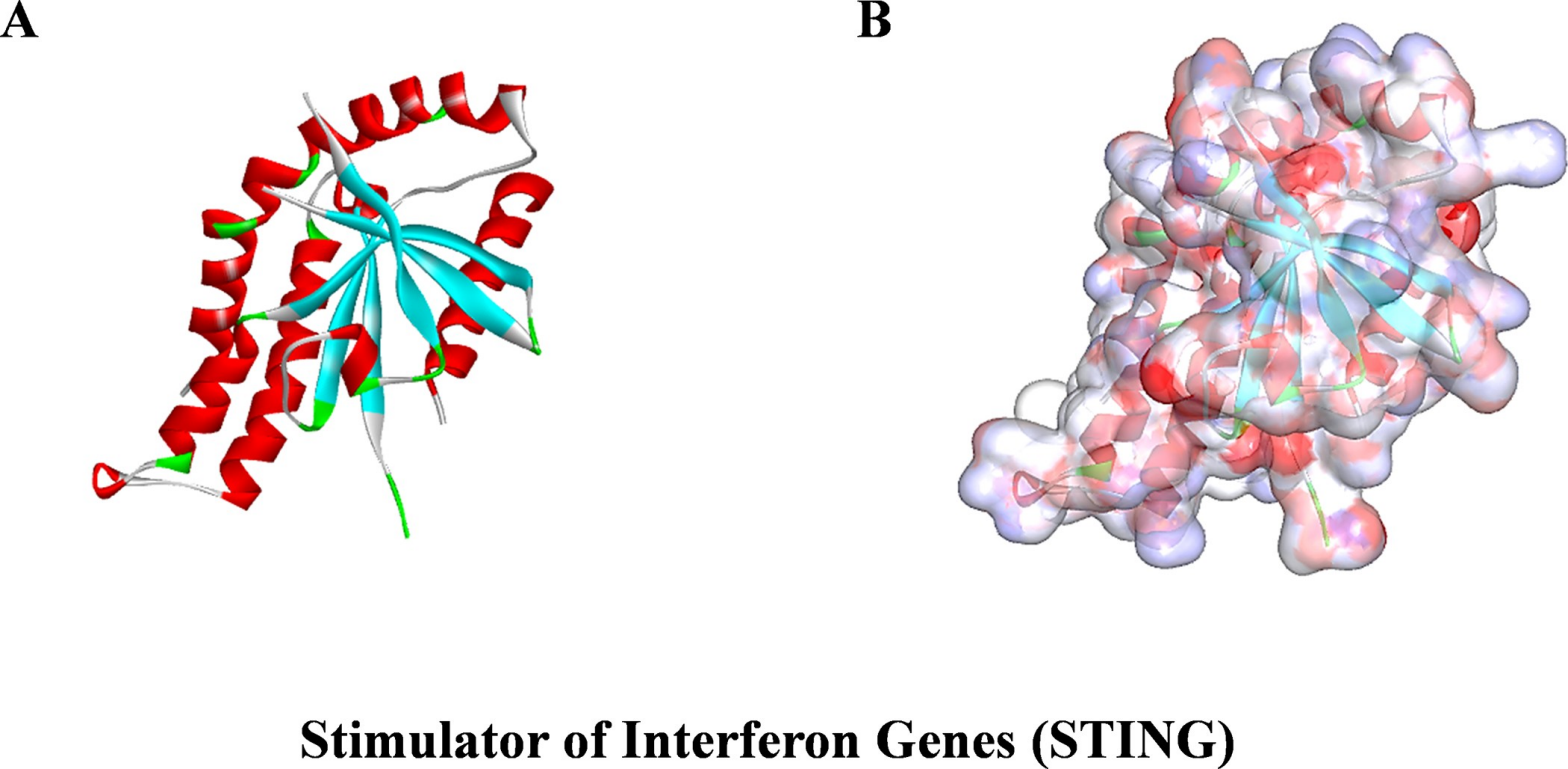
The molecular structure of STING. Initial molecular structure was shown in **(A)**, and surface of binding area were added in **(B)**, blue represented positive charge, red represented negative charge.

Ligand-binding pocket region of STING was selected as the binding site to identify new compounds that could potentially stimulate STING. A virtual screening was carried out using libdock module of Discovery Studio 4.5 [24]. Libdock (San Diego, CA, USA) is a rigid-based docking program. It calculates hotspots for the protein using a grid placed into the binding site and also using polar and apolar probes. Then, the hotspots are further used to align the ligands to form favorable interaction. The Smart Minimiser algorithm and CHARMm force field (Cambridge, MA, USA) were performed for ligands minimization. After minimized, all the ligand poses were ranked based on the ligands score. The 2.45 Å crystal structure of STING in complex with amidobenzimidazole (ABZI) (PDB ID: 6DXL) [11] was downloaded from protein data bank (PDB) and imported to the working environment of libdock. The chemical structure of STING was shown in **Fig 1**. The protein was prepared by removing crystal water and other hetero-atoms, followed by addition of hydrogen, protonation, ionization and energy minimization. The CHARMm force field and the Smart Minimiser algorithm were applied for energy minimization [25]. The minimization performed 2000 steps with an RMS (Root Mean Square) gradient tolerance of 0.1, and the final RMS gradient was 0.09778. The prepared protein was used to define the binding site, ABZI binding site was selected as the active sites for docking. Virtual screening was carried out by docking all the prepared ligands at the defined active site using libdock. Based on the libdock score, all the docked poses were ranked and grouped by the compounds’ name.

### ADME (Absorption, Distribution, Metabolism, Excretion) and toxicity prediction

ADME module of Discovery Studio 4.5 was employed to calculate the absorption, distribution, metabolism, excretion of selected compounds. TOPKAT (Toxicity Prediction by Komputer Assisted Technology) modules of DS4.5 was also employed to calculate the toxicity and other properties of all the potential compounds, including their aqueous solubility, blood-brain barrier (BBB) penetration, cytochrome P450 2D6 (CYP2D6) inhibition, hepatotoxicity, human intestinal absorption, plasma protein binding (PPB) level, rodent carcinogenicity, ames mutagenicity and developmental toxicity potential. These pharmacological properties were fully considered when selecting drug candidates for STING.

### Molecular docking and MM/GBSA calculation

CDOCKER and MM/GBSA module of Discovery Studio 4.5 was used for molecular docking study. CDOCKER is a molecular docking method based on CHARMm36 force field, which can produce high-precision docking results. The receptor is held rigid while the ligands are allowed to flex during the docking process. For each complex pose, the CHARMm energy (interaction energy plus ligand strain) and the interaction energy, which indicated ligand binding affinity, were calculated. Molecular Mechanics with Generalized Born and Surface area solvation (MM/GBSA) was conducted to verify the free energy of ligand-STING complex. Crystal structure of STING was obtained from the protein data bank. The crystal water molecules were generally removed in rigid and semi-flexible docking process [26,27], since the fixed water molecules might affect the formation of receptor-ligand complex. Next, the water molecules were removed and hydrogen atoms were added to the protein. In order to prove the reliability of the combination mode, the initial compound ABZI was extracted from the binding site and then re-docked into the crystal structure of STING. The CHARMm36 force field was used for both receptors and ligands. The binding site sphere of STING was defined as the region that came within radius 16 Å from the geometric centroid of the ligand ABZI. During the docking process, the ligands were allowed to bind to the residues within the binding site spheres. The structures of identified hits were prepared and docked into the binding pocket of STING. The CDOCKER process was performed. Ten docking poses were generated for each ligand and the best pose was selected based on high docking scores and appropriate docking orientations [28,29]. Different poses of each test molecules were generated and analyzed on the basis of CDOCKER interaction energy, MM/GBSA free energy, respectively.

### Molecular dynamics simulation

The best binding conformations of each compounds-STING complex were selected and prepared for molecular dynamics simulation. The ligand-receptor complex was put into an orthorhombic box and solvated with an explicit periodic boundary solvation water model. In order to simulate the physiological environment, solidum chloride were added to the system with the ionic strength of 0.145. Then, the system was subjected to the CHARMm forcefield and relaxed by energy minimization (500 steps of steepest descent and 500 steps of conjugated gradient), with the final RMS gradient of 0.289. The system was slowly driven from an initial temperature of 50 K to the target temperature of 300 K for 200 ps and equilibration simulations were run for 250 ps. Molecular dynamics simulations (production) were performed for 1 ns with time step of 1 ps. The simulation was performed with the NPT (normal pressure and temperature) system at a constant temperature of 300 K. The particle mesh ewald (PME) algorithm was used to calculate long range electrostatics, and the linear constraint solver (LINCS) algorithm was adapted to fix all bonds involving hydrogen. With initial complex setting as a reference, the trajectory was determined for structural properties, root mean-square deviation (RMSD), and potential energy by using trajectory protocol in Discovery Studio 4.5 (San Diego, CA, USA).

### Animal experiments to verify effectiveness of the compound

30 nude mice and compounds 1–3 were obtained from the Animal Experiment base in clinical college of Jilin University, and experimental protocols were approved by Jilin University Ethics Committee. A total of 10^5^ Colon adenocarcinioma 26 cells per 100 μl PBS were injected subcutaneously into the right flank of each mouse to establish tumors. Mice were divided into four groups: (a) control group with tumor cells injection; (b, c, d) treatment groups with tumor injection and treated by compound 1,2,3 at dosage of 10 mg/kg, respectively. Four hours after injection of Colon adenocarcinioma 26 cells, compound 1,2,3 were intravenously injected into the tumor-bearing mice through tail daily, 20 days total. Tumor volumes were measured and weight of tumor-bearing mice were checked daily. Each compound was injected into 7 mice, and final results were averaged to show representative data. On day 20^th^, check amounts of survival mice, then all mice were measured and sacrificed, and all the tumors were removed and weighed.

## Results

### Virtual screening of natural products database against stimulator of interferon genes (STING)

Ligand-binding pocket was an important regulatory site of STING, cGAMP bound to this pocket region to activate the function of STING in normal physical environment, therefore, this pocket region was selected as a reference site. A total of 17776 purchasable natural named product molecules were taken from the ZINC15 database. Molecule structure of STING (PDB ID: 6DXL) was selected as the receptor protein. ABZI, one of STING agonists, was chosen as a reference compound to evaluate the binding ability of other compounds. 2893 compounds were identified to bind with STING stably by libdock algorithm. Among these compounds, 67 compounds had higher libdock scores than ABZI (Libdock score: 108.6, ranking: 68). The top 20 ranked compounds based on libdock scores were listed in **Table 1**.

**Table 1:** Top 20 ranked compounds with higher Libdock scores than ABZI.

Number	Compounds	Libdock score	Number	Compounds	Libdock score
1	ZINC000053147179	137.474	11	ZINC000015122269	121.723
2	ZINC000015149223	133.101	12	ZINC000011616636	121.484
3	ZINC000011616633	132.88	13	ZINC000042805135	120.903
4	ZINC000003938684	131.996	14	ZINC000049088142	120.538
5	ZINC000005601526	130.832	15	ZINC000028820378	119.594
6	ZINC000049784088	127.577	16	ZINC000085826837	119.401
7	ZINC000001577210	126.986	17	ZINC000028968101	119.262
8	ZINC000072133963	124.764	18	ZINC000006845904	118.641
9	ZINC000017654900	122.373	19	ZINC000040866224	118.631
10	ZINC000004095521	121.757	20	ZINC000096023886	118.585

### ADME (Absorption, Distribution, Metabolism, Excretion) and toxicity prediction

Pharmacological properties of all selected ligands and ABZI were first predicted by ADME module of Discovery Studio 4.5, including brain/blood barrier (BBB), human intestinal absorption, aqueous solubility, cytochrome P450 2D6 (CYP2D6) binding, hepatotoxicity and plasma protein binding properties (PPB) (**Table 2**). The aqueous solubility prediction (defined in water at 25°C) indicated that all the compounds were soluble in water. For human intestinal absorption, 5 compounds and ABZI had a good absorption level and 8 compounds had a moderate absorption level. 10 compounds were found to be highly bound with plasma protein and the rest were just opposite. All compounds were predicted to be non-inhibitors of cytochrome P450 2D6 (CYP2D6) except ZINC000053147179, ZINC000028968101 and ZINC000006845904, which was one of the important enzymes involved in drug metabolism. For hepatotoxicity, 13 compounds were predicted as non-toxic, which was similar to ABZI.

**Table 2:** ADME (Adsorption, Distribution, Metabolism, Excretion) properties of compounds.

Number	Compounds	Solubility Level ^a^	BBB Level ^b^	CYP2D6 ^c^	Hepatotoxicity ^d^	Absorption Level ^e^	PPB Level ^f^
1	ZINC000053147179	1	4	1	1	0	1
2	ZINC000015149223	1	4	0	0	0	1
3	ZINC000011616633	1	4	0	0	0	1
4	ZINC000003938684	2	2	0	1	1	1
5	ZINC000005601526	2	4	0	0	1	0
6	ZINC000049784088	1	2	0	0	2	0
7	ZINC000001577210	3	4	0	0	0	1
8	ZINC000072133963	1	4	0	0	1	1
9	ZINC000017654900	1	2	0	1	2	0
10	ZINC000004095521	1	4	0	0	3	1
11	ZINC000015122269	2	1	0	1	1	1
12	ZINC000011616636	2	4	0	0	2	0
13	ZINC000042805135	1	3	0	1	2	0
14	ZINC000049088142	1	2	0	1	1	0
15	ZINC000028820378	3	2	0	0	1	0
16	ZINC000085826837	1	4	0	0	1	0
17	ZINC000028968101	1	4	1	1	1	1
18	ZINC000006845904	2	4	1	0	3	1
19	ZINC000040866224	2	2	0	0	2	0
20	ZINC000096023886	3	4	0	0	0	0
21	amidobenzimidazole	2	4	0	1	2	0

^a^ Aqueous-solubility level: 0 (extremely low); 1 (very low, but possible); 2 (low); 3 (good)

^b^ Blood Brain Barrier level: 0 (Very high penetrant); 1 (High); 2 (Medium); 3 (Low); 4 (Undefined)

^c^ Cytochrome P450 2D6 level: 0 (Non-inhibitor); 1 (Inhibitor)

^d^ Hepatotoxicity: 0 (Nontoxic); 1 (Toxic)

^e^ Human-intestinal absorption level: 0 (good); 1 (moderate); 2 (poor); 3 (very poor)

^f^ Plasma Protein Binding: 0 (Absorbent weak); 1 (Absorbent strong)

**Fig 2:**
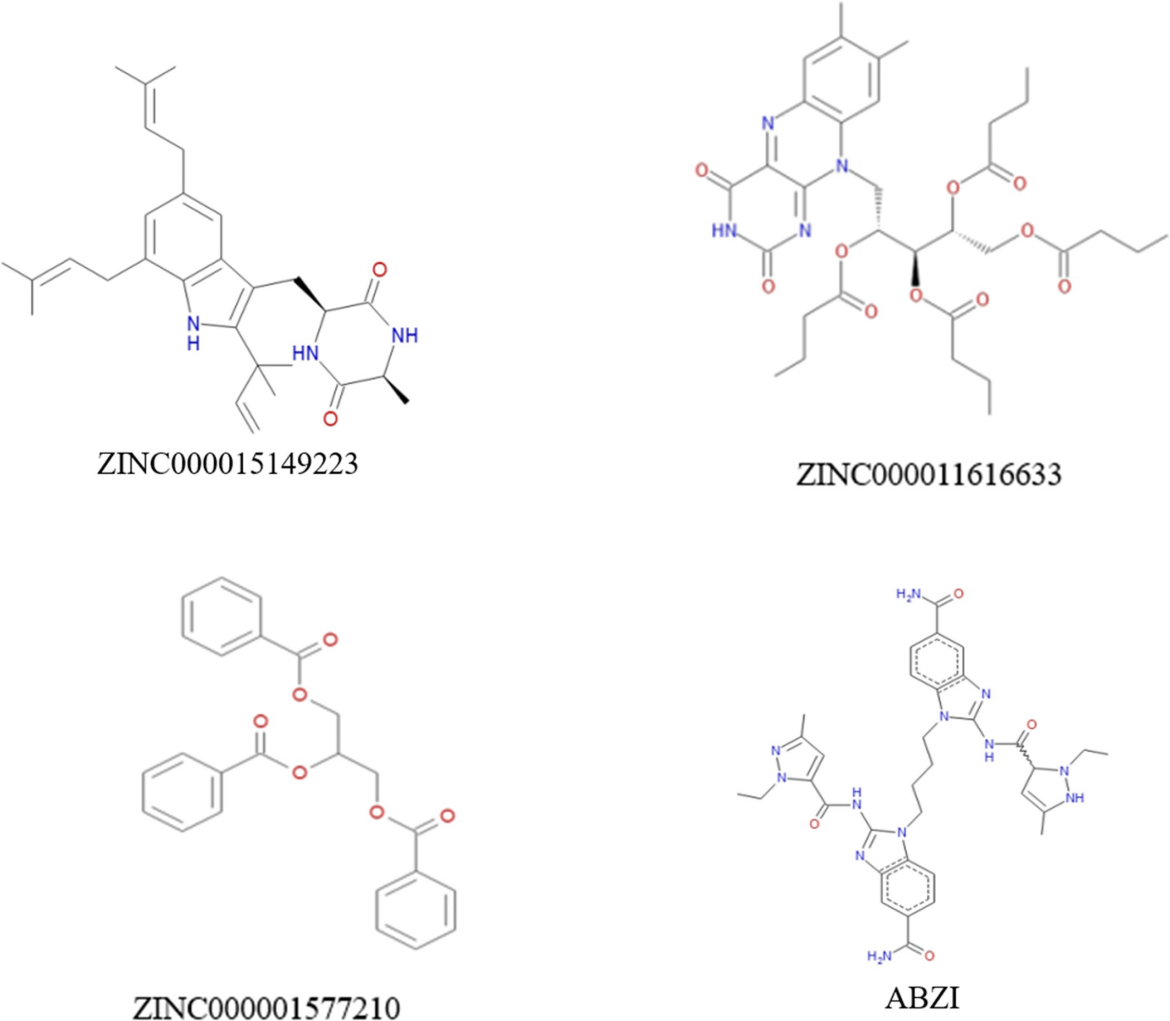
The structures of ABZI and novel compounds selected from virtual screening.

Safety was also fully investigated in this study. To examine safety of the selected compounds, different toxicity indicators of the compounds and ABZI, including Ames mutagenicity (AMES), Rodent carcinogenicity (based on the U.S. National Toxicology Program (NTP) dataset) and developmental toxicity potential (DTP) properties, were predicted using TOPKAT module of Discovery Studio 4.5 (**Table 3**). Results showed that 9 compounds had non-developmental toxicity potential. Considering all the results above, compound 1(ZINC000015149223), compound 2(ZINC000011616633) and compound 3(ZINC000001577210) were identified as ideal lead compounds, which were not CYP2D6 inhibitors thereby without hepatotoxicity. Moreover, they were predicted with less ames mutagenicity, rodent carcinogenicity and developmental toxicity potential compared with other compounds, which also strongly suggested their perspective application in drug development. According to **Fig 2**, these three compounds and the reference compound ABZI were found to be highly axisymmetric in their structures, which were similar to the structure of cGAMP. After analyzing the molecular formula of these four compounds, ABZI was found to be formed a bridge by two monomers which connecting STING dimers. The other three natural compounds selected in study were inborn axisymmetric, and they don’t have to form a dimer to perform their functions. In summary, compounds 1–3 were identified as safe drug candidates and selected for following research (**Fig 2**).

**Table 3:** Toxicities of compounds.

Number	Compounds	Mouse NTP ^a^		Rat NTP ^a^		AMES ^b^	DTP ^c^
		Female	Male	Female	Male		
1	ZINC000053147179	1	1	1	0	0	1
2	ZINC000015149223	1	0.017	1	0	0	0.205
3	ZINC000011616633	0	1	0	1	0.020	0
4	ZINC000003938684	0.025	0.953	1	0.026	0	1
5	ZINC000005601526	0.331	1	0.115	1	0	0.700
6	ZINC000049784088	0.995	0	0	0.008	1	1
7	ZINC000001577210	0	0.173	0	0.952	0	0.040
8	ZINC000072133963	0.208	0	1	1	0	0.007
9	ZINC000017654900	1	0	0.816	0	0	0.152
10	ZINC000004095521	0	1	1	0	0.017	0
11	ZINC000015122269	0.975	0	0.006	0.959	0.001	1
12	ZINC000011616636	0	1	1	1	1	1
13	ZINC000042805135	1	0	1	0.979	0	1
14	ZINC000049088142	0	1	1	0	0	0
15	ZINC000028820378	0.197	0	1	0.09	0.992	1
16	ZINC000085826837	0.186	1	1	0.998	0	1
17	ZINC000028968101	1	0.021	0.060	0.997	1	1
18	ZINC000006845904	0	1	1	0	0.014	0
19	ZINC000040866224	0.001	0.144	0.004	0	0	1
20	ZINC000096023886	0	0.372	1	0.997	0.830	1
21	amidobenzimidazole	1	1	0	0.344	1	0.039

^a^ <0.3 (Non-Carcinogen); >0.7 (Carcinogen)

^b^ <0.3 (Non-Mutagen); >0.7 (Mutagen)

^c^ <0.3 (Non-Toxic); >0.7 (Toxic)

### Ligand binding analysis

**Fig 3:**
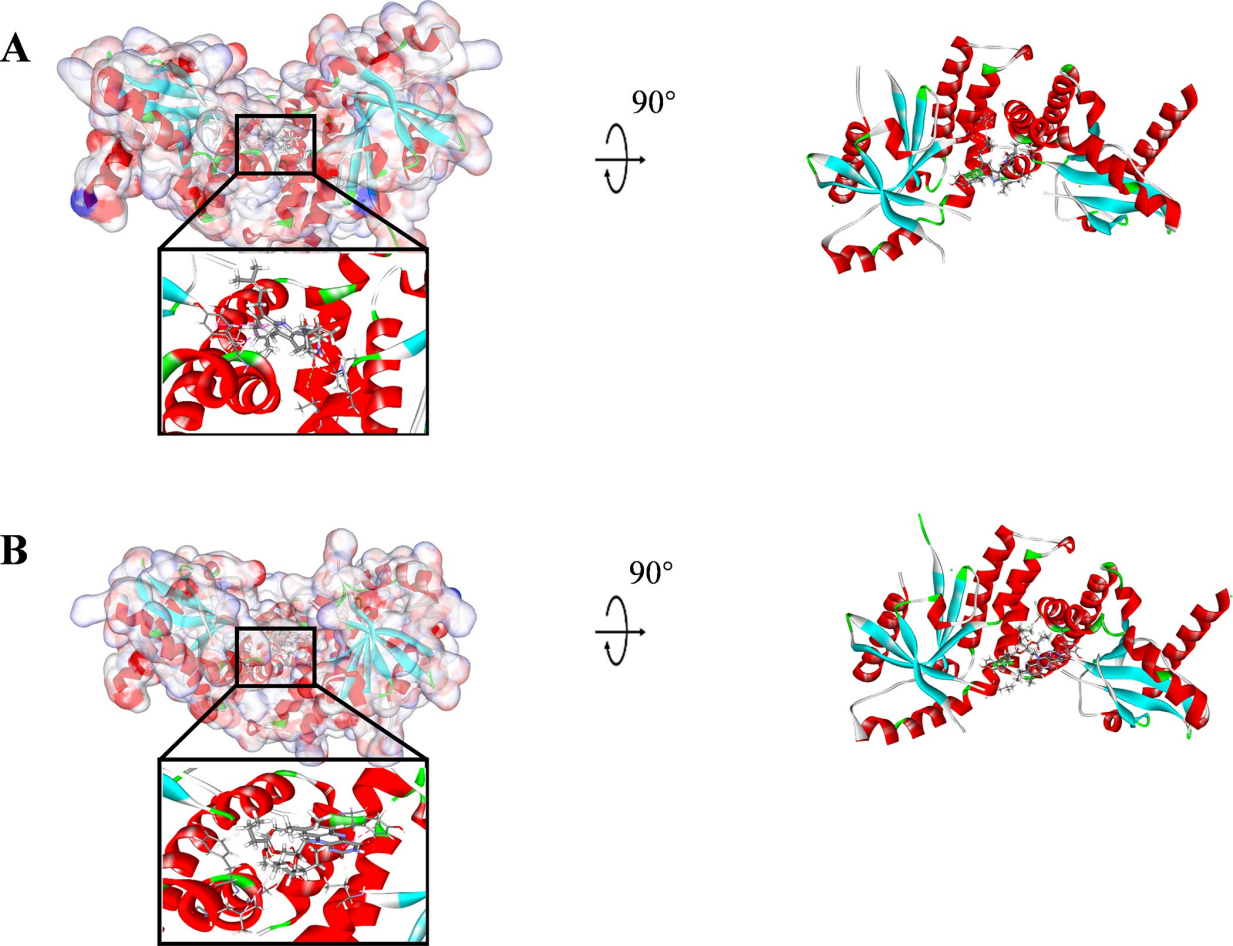
Schematic drawing of interactions between ligands and STING, the surface of binding area were added, blue represented positive charge, red represented negative charge, and ligands were shown in sticks, the structure around the ligand-receptor junction were shown in thinner sticks. (**A**) ZINC000015149223-STING complex; (**B**) ZINC000011616633-STING complex.

**Fig 4:**
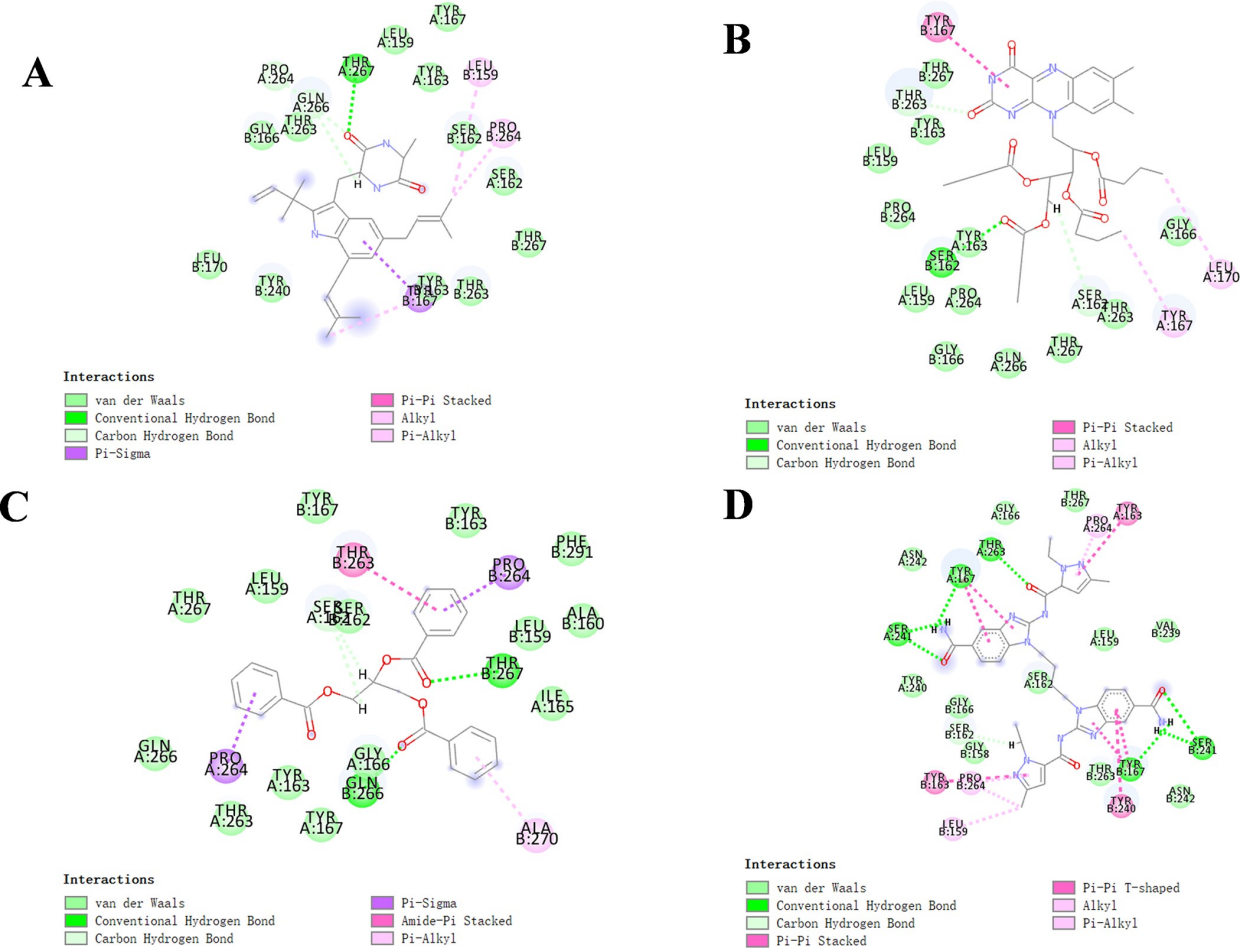
The inter-molecular interaction of the predicted binding modes of (**A**) ZINC000015149223 to STING; (**B**) ZINC000011616633 to STING, (**C**) ZINC000001577210 to STING and (**D**) ABZI to STING.

The RMSD (Root Mean Square Deviation) between the docked pose and the crystal structure of the complex was 0.6 Å, indicating the CDOCKER module applied in this study was highly reliable. Compounds 1–3 were docked into the molecule structure of STING by CDOCKER module under CHARMm36 force field, CDOCKER potential energy and MM/GBSA binding free energy were calculated and displayed in **Tables 4** and **5**. Results showed that the CDOCKER potential energy of compound 1, compound 2 and compound 3 were significant lower than the reference ligand ABZI (-41.8047kcal/mol), MM/GBSA binding free energy also calculated that these three compounds contributed lower energy compared to the reference ligand ABZI (-82.0470kcal/mol), which indicated that these three compounds may have a higher binding affinity with STING compared to ABZI. Structural computation was also performed for the hydrogen bonds and Pi-Pi interactions of ligands-STING complex (As shown in **Fig 3, S1 Fig**, **Fig 4** and **Tables 6** and **7**). Results showed that compound 1 formed one pair of hydrogen bonds with STING, by the O17 of compound and THR267:HG1 of STING. Also, one pair of pi-pi interaction was formed in the complex. Compound 2 formed one pair of hydrogen bond, by the O5 of the compound and SER162:HG of STING. There was also one pair of pi-pi interaction formed within the complex. Compound 3 formed two pairs of hydrogen bonds with STING, one is from the O1 of compound to GLN266:HE21 of STING, the other one is from O18 of the compound to THR267:HG1 of STING. No pi-pi interactions were observed. For the reference compound ABZI, it formed seven hydrogen bonds with STING, (A:SER241:HN-ABZI:O88, A:SER241:O-ABZI:H86, A:THR263:HG1-ABZI:O19, A:TYR167:OH-ABZI:H87, B:SER241:HN-ABZI:O83, B:SER241:O-ABZI:H81, B:TYR167:OH-ABZI:H82, respectively). It also formed seven pairs of pi-pi interactions with STING.

**Table 4:** CDOCKER potential energy of compounds with stimulator interferon genes (STING) under CHARMm36 force field.

compounds	CDOCKER potential energy (Kcal/mol)
ZINC000015149223	-49.0339
ZINC000011616633	-54.8919
ZINC000001577210	-43.0851
ABZI	-41.8047

**Table 5:** MM/GBSA binding free energy of compounds with stimulator interferon genes (STING).

compounds	MM/GBSA energy (Kcal/mol)
ZINC000015149223	-93.6319
ZINC000011616633	-96.0275
ZINC000001577210	-101.8364
ABZI	-82.0470

**Table 6:** Hydrogen bond interaction parameters for each compound and STING residues.

Receptor	Compound	Donor Atom	Receptor Atom	Distances (Å)
STING	ZINC000015149223	A:THR267:HG1	ZINC000015149223:O17	2.34
	ZINC000011616633	B:SER162:HG	ZINC000011616633:O5	2.51
	ZINC000001577210	B:GLN266:HE21	ZINC000001577210:O1	2.59
		B:THR267:HG1	ZINC000001577210:O18	1.63
	ABZI	A:THR263:HG1	ABZI:O19	2.50
		A:TYR167:OH	ABZI:H87	2.54
		A:SER241:O	ABZI:H86	1.91
		A:SER241:HN	ABZI:O88	2.34
		B:SER241:HN	ABZI:O83	2.75
		B:SER241:O	ABZI:H81	2.06
		B:TYR167:OH	ABZI:H82	2.54

**Table 7:** Pi-Pi interaction parameters for each compound and STING residues.

Receptor	Compound	End 1	End 2	Distance (Å)
STING	ZINC000015149223	B:TYR167	ZINC000015149223	5.28
	ZINC000011616633	B:TYR167	ZINC000011616633	4.63
	ZINC000001577210	Ng	Ng	Ng
	ABZI	A:TYR163	ABZI	5.88
		A:TYR167	ABZI	5.09
		A:TYR167	ABZI	4.72
		B:TYR163	ABZI	5.97
		B:TYR240	ABZI	5.13
		B:TYR167	ABZI	4.72
		B:TYR167	ABZI	5.03

Ng: Not given.

### Molecular dynamics simulation

**Fig 5:**
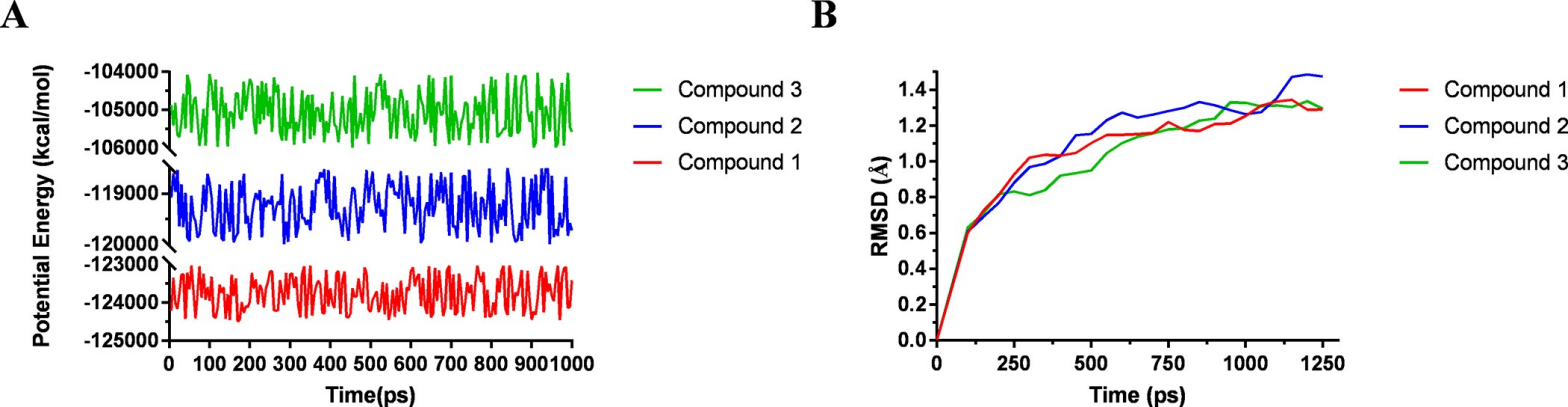
Results of molecular dynamics simulation of three complexes. (**A**) Potential Energy; (**B**) Average backbone RMSD.

To evaluate the stabilities of ligand-STING complexes under natural environmental conditions, molecular dynamics simulation was conducted. The RMSD curves and potential energy profiles of each complex were shown in **Fig 5**. The trajectories of complexes reached equilibrium after 200 ps, RMSD and potential energy of the complexes got stabilized with time. Molecular dynamics simulation results validated that these hydrogen bonds and pi-pi interactions formed by compounds and STING contributed to the stability of the complexes. Results showed that these three compounds interacted with STING, their complex could exist in natural environment steadily and have modulatory effects on STING as ABZI did.

### Experimental results to validate the effectiveness of the compounds

**Fig 6:**
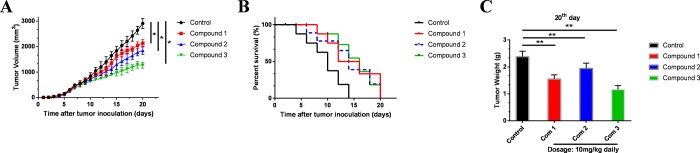
Animal experiments to against tumor activity. Tumor-bearing mice were treated with compound 1,2,3 at dosage of 10 mg/kg, respectively. (**A**) Mean tumor volumes. (**B**) Survival percentage of Mice. (**C**) Tumor weights on 20^th^ day. Data were represented as Mean ± SEM, *p<0.05 and **p<0.01.

Animal experiments were conducted to validate effectiveness of these selected compounds (**Fig 6**), results showed that these three compounds had effect to suppress tumor growth, among which compound 3 contributed most to the efficiency. After 9^th^ day, compounds 1–3 played a significant role in killing tumor. On 20^th^ day, tumor volume in control group was 2920 mm^3^, the tumor volumes after drug treatment by compounds 1–3 were 2110, 1850, 1440 mm^3^, respectively. Data were represented as mean ± SEM and p<0.05. Survival percent chart illustrated that compounds 1–3 had effect in prolonging survival period. Tumor weight chart also demonstrated that on 20^th^ day, final tumor weight in control group was 2.38g, compared with tumor weight after treatment by compounds 1–3 were 1.55g, 1.94g, 1.16g (mean ± SEM, p<0.01), respectively.

## Discussion

cGAS-cGAMP-STING pathway plays a significant role in host defense against viral and bacterial infection. Activation of STING elicits a type-I interferon response, which propagates interferon receptor signaling in tumor-resident dendritic cells and leads to anti-tumor CD8+ T cell responses in vivo, resulting in a correspondent immune response to eliminate cancer or bacteria. However, due to shortage of tumor-specific T cells or inadequate activation of STING protein, cGAS-cGAMP-STING pathway can be suppressed in some serve diseases, such as infection and cancer [9]. Therefore, it’s of great importance to re-stimulate cGAS-cGAMP-STING pathway and promote T-cell proliferation in cancer immunotherapy. Nonetheless, it is very difficult to stimulate STING and turn on immune pathway by artificial methods. Previous researches hoped to reactivate STING pathway by perform intravenous injection with high doses of cGAMP daily, however, it only resulted in modest in-vivo efficacy [12]. Other study showed that intramuscular injection of cGAMP can delay tumor growth after tumor implantation, whereas titration experiment showed that cGAMP enhanced its effect mainly depended on dose [13], so far it’s not practical in clinical application because the cost is high and cGAMP tend to decompose in natural environment. Additionally, cGAMP contains two phosphodiester bonds, which hinder its ability to permeate cells [13]. Currently, most researches focused on pathological role and molecular biological role of cGAS-cGAMP-STING pathway, rather than how to stimulate the STING protein directly by small molecular agonists. Although great progress of compounds have been made regarding to STING drug design and development, only one agonist ABZI, which is selected as a reference drug in this study, has shown a perspective therapeutic effect until now. Therefore, there is an urgent need to screen more compounds targeting STING for clinical application.

In this study, four modules of Discovery Studio 4.5, including Libdock, ADME/TOPKAT, CDOCKER and Molecular Dynamics Simulation, were employed to screen and analyze the structural biological properties of novel potential compounds, respectively. Molecular conformation, pharmacological properties, binding affinity and stability were also fully analyzed to determine superiority of the selected compounds. 17776 purchasable, natural, named product molecules were obtained from the ZINC15 database for virtual screening. Libdock score represented degree of energy optimization and stability of the conformation. Compounds with a high libdock score illustrated that it had a pretty energy optimization and a stable conformation compared with others. After calculated by libdock module of Discovery Studio 4.5, 2893 compounds were identified to have a high binding affinity with STING. Among these compounds, 67 compounds had higher libdock scores than the reference compound ABZI (Libdock score: 108.6, ranking: 68), which indicated that these 67 compounds could combine with STING and form a more stable conformation with better energy optimization compared to ABZI. The top 20 natural compounds were selected based on libdock score and pooled into further study.

ADME (absorption, distribution, metabolism, excretion) and toxicity properties of the obtained compounds were carried out to evaluate the pharmacological properties of these selected compounds. Results showed that compounds 1–3 were identified as ideal lead compounds. Since they were all soluble in water and also had a good absorption level. Meanwhile, they were non-inhibitors of cytochrome P450 2D6 (CYP2D6), which indicated they didn’t have hepatotoxicity. Additionally, these three compounds were also predicted with less ames mutagenicity, rodent carcinogenicity and developmental toxicity potential compared with other compounds, which also strongly suggested their perspective application in drug development. On the other hand, the rest drug in the list also had potential application in drug development even though they possessed toxicity, since specific groups and atoms could be added to reduce its toxicity. Considering all the results above, compounds 1–3 were selected as ideal lead compounds and further analysis were carried out.

Binding mechanism and chemical bonds of the selected candidate compounds were also investigated. CDOCKER module computation demonstrated that CDOCKER interaction energy of compounds 1–3 were significant lower than the reference ligand ABZI (-41.8047kcal/mol), MM/GBSA binding free energy also calculated that these three compounds contributed lower energy compared to the reference ligand ABZI (-82.0470kcal/mol), which indicated that these three compounds may have a higher binding affinity with STING compared to ABZI. Next, the chemical structures and binding mechanism of these compounds were analyzed by molecular structural inspection, results indicated that these three products and the reference compound ABZI were found to be highly axisymmetric in their structures, which were very similar to the chemical structure of cGAMP. ABZI was found that under catalysis of liver processing enzyme, ABZI dimer was formed by two monomers and then connect STING dimers as a bridge. While the three natural compounds selected in study were inborn axisymmetric, they don’t have to be form dimers by in-vivo processing. Therefore, they may possess a favorable agonist effect with less by-products and toxicity compared to ABZI.

Next, their stabilities were also assessed by performing molecular dynamics simulation. RMSD and potential energy of these ligand-STING complexes were calculated, results showed that the trajectories of complexes reached equilibrium after 200 ps, RMSD and potential energy of the complexes got stabilized with time, which illustrated that these three complexes could exist in natural environment stably. Based on these results, modification and refinement could be perspectively carried out to make ligand and receptor bind more firmly, acting as a bridge connecting two STING proteins.

Finally, animal experiment was conducted to validated the effectiveness of these selected compounds, and these compounds were demonstrated that they played a role in killing tumor after 9^th^ day. Survival percent chart illustrated that compounds 1–3 had effect in prolonging survival period, which may contribute to live quality. Although these drugs were not powerful enough compared to drugs [12], [13], it is worth to know that this study aimed to screen and identify ideal lead compounds which had potential regulatory functions to STING. Different groups could be added on this skeleton to make the drug more efficient. After elaborate medication design and refinement, they could be better potential agonists.

It’s also worth noting that the compounds studied in our research mainly focused on the development of agonists, but they are also of great importance in STING inhibitors development. Agonist and inhibitor usually share similar skeleton in chemical structure, the opposite effects are mainly produced by adding different groups or atoms. With the advantage of their innate affinity for STING, natural compounds identified in this study could provide valuable resource for STING related drugs development.

Although this study was conducted by elaborate design and precise measurements have been performed, we still admitted that there are still some limitations in this study. More experiments need to be performed to validate our results and more indicators regarding to drug safety, such as MTD (Maximum Tolerated Dosage) and AB (Aerobic Biodegradability) measurements, should also be assessed in our future study.

## Conclusions

This study employed a series of structural biological and chemistry method (including virtual screening, molecule docking and etc) to screen and identify the lead compounds which had potential regulatory functions to STING. In summary, compounds 1–3 were potential agonists targeting STING protein. These compounds were safe drug candidates and had a great significance in STING agonists development. Additionally, this study provided a list of drug candidates and their pharmacological properties, which could provide a solid basis for STING agonists development research.

## Supporting information

S1 Fig Schematic drawing of interactions between ligands and STING, the surface of binding area were added, blue represented positive charge, red represented negative charge, and ligands were shown in sticks, the structure around the ligand-receptor junction were shown in thinner sticks. (**A**) ZINC000001577210-STING complex; (**B**) ABZI-STING complex.
(TIF)

## Abbreviations

ADME: Absorption, Distribution, Metabolism, Excretion
ABZI: amidobenzimidazole.
Compound 1: ZINC000015149223
Compound 2: ZINC000011616633
Compound 3: ZINC000001577210
CYP2D6: Cytochrome P450 2D6
BBB: Blood-brain barrier
DS: Discovery Studio
DTP: Developmental toxicity potential
MM/GBSA: Molecular Mechanics Generalized Born Surface Area
PPB: Plasma protein binding
RMS: Root Mean Square
RMSD: Root Mean-Square Deviation
STING: Stimulator of Interferon Genes
