# Supplementary figures and images for: Computational study on new natural compound agonists of stimulator of interferon genes (STING)
Source: PLoS One. 2019 May 23;14(5):e0216678. doi: 10.1371/journal.pone.0216678 (PMC6532845; doi:10.1371/journal.pone.0216678)

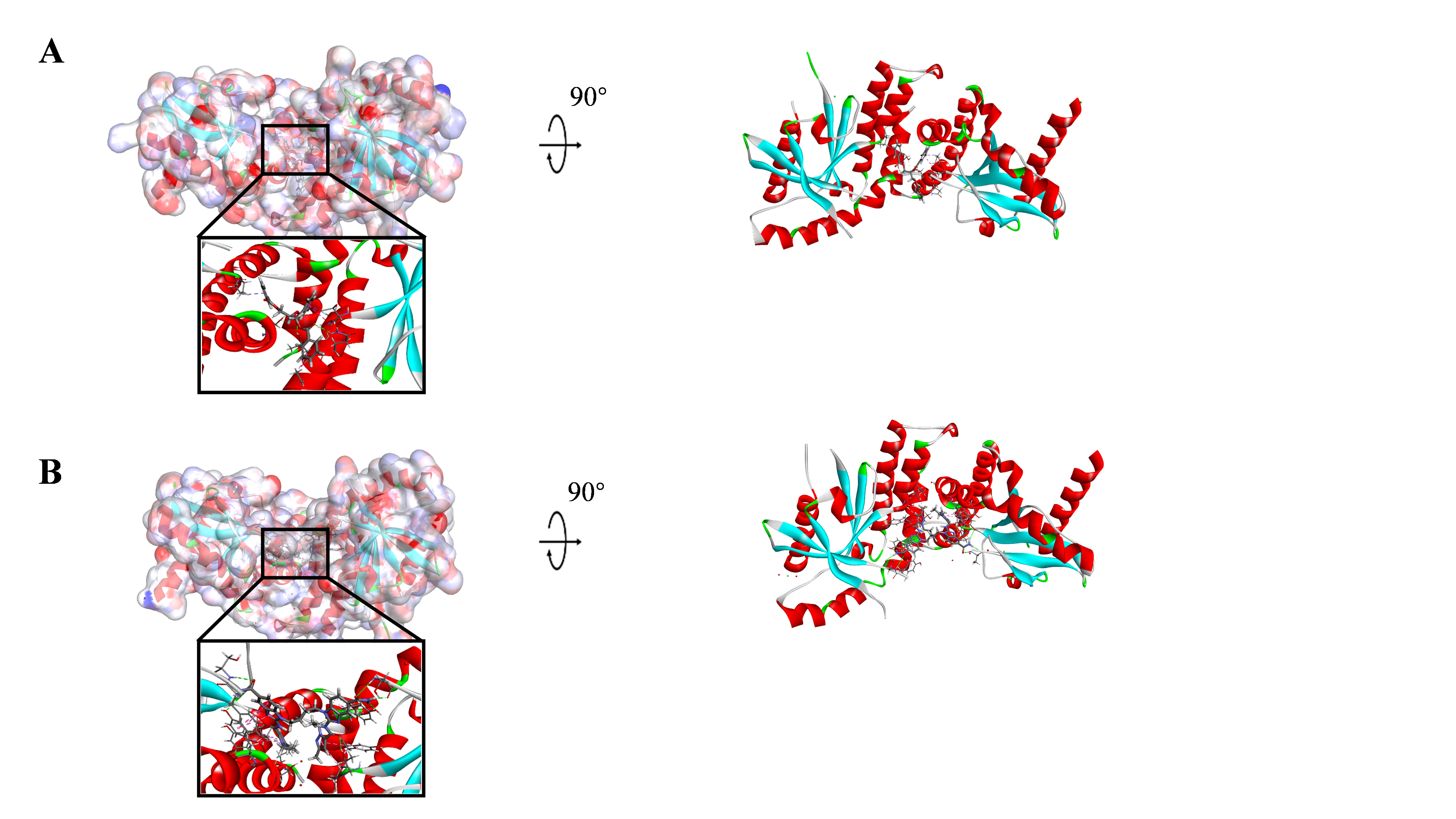

Supplement: S1 Fig — (A) ZINC000001577210-STING complex; (B) ABZI-STING complex. (TIF) [file pone.0216678.s001.tif]
